# Evaluation of physicians' professional performance: An iterative development and validation study of multisource feedback instruments

**DOI:** 10.1186/1472-6963-12-80

**Published:** 2012-03-26

**Authors:** Karlijn Overeem, Hub C Wollersheim, Onyebuchi A Arah, Juliette K Cruijsberg, Richard PTM Grol, Kiki MJMH Lombarts

**Affiliations:** 1 IQ healthcare, Radboud University Nijmegen Medical Centre, Nijmegen, The Netherlands; 2 Department of Epidemiology, School of Public Health, University of California, Los Angeles (UCLA), Los Angeles, California, USA; 3 Center for Health Policy Research, UCLA, Los Angeles, California, USA; 4 Department of Quality and Process Innovation, Academic Medical Centre, University of Amsterdam, Amsterdam, The Netherlands

## Abstract

**Background:**

There is a global need to assess physicians' professional performance in actual clinical practice. Valid and reliable instruments are necessary to support these efforts. This study focuses on the reliability and validity, the influences of some sociodemographic biasing factors, associations between self and other evaluations, and the number of evaluations needed for reliable assessment of a physician based on the three instruments used for the multisource assessment of physicians' professional performance in the Netherlands.

**Methods:**

This observational validation study of three instruments underlying multisource feedback (MSF) was set in 26 non-academic hospitals in the Netherlands. In total, 146 hospital-based physicians took part in the study. Each physician's professional performance was assessed by peers (physician colleagues), co-workers (including nurses, secretary assistants and other healthcare professionals) and patients. Physicians also completed a self-evaluation. Ratings of 864 peers, 894 co-workers and 1960 patients on MSF were available. We used principal components analysis and methods of classical test theory to evaluate the factor structure, reliability and validity of instruments. We used Pearson's correlation coefficient and linear mixed models to address other objectives.

**Results:**

The peer, co-worker and patient instruments respectively had six factors, three factors and one factor with high internal consistencies (Cronbach's alpha 0.95 - 0.96). It appeared that only 2 percent of variance in the mean ratings could be attributed to biasing factors. Self-ratings were not correlated with peer, co-worker or patient ratings. However, ratings of peers, co-workers and patients were correlated. Five peer evaluations, five co-worker evaluations and 11 patient evaluations are required to achieve reliable results (reliability coefficient ≥ 0.70).

**Conclusions:**

The study demonstrated that the three MSF instruments produced reliable and valid data for evaluating physicians' professional performance in the Netherlands. Scores from peers, co-workers and patients were not correlated with self-evaluations. Future research should examine improvement of performance when using MSF.

## Background

In view of demands for high quality care, many health care systems aim to assess physicians' professional performance. As the ability to self-assess has shown to be limited, there is a need for external assessments [1]. Reliable, valid, feasible and effective measures of performance are vital to support these efforts. Since 1993, multisource feedback (MSF) or 360-degree evaluation is increasingly used in health systems around the world as a way of assessing multiple components of professional performance. MSF involves external evaluation of physicians' performance on various tasks by: 1) peers with knowledge of a similar scope of practice, 2) non-physician co-workers (nurses, allied healthcare professionals or administrative staff) and 3) patients [2]. Raters in those three categories are those who observed the physician's behaviour in order to be able to answer questions about a physician's performance. Physicians also complete a questionnaire about their own performance and these ratings are compared with others' ratings in order to examine directions for change [3]. Before the widespread use of MSF is merited, it is of vital importance that physicians, managers and patients have confidence in the validity and reliability of instruments applied in MSF [4]. In Canada and the United Kingdom, the reliability and validity of instruments used for MSF have been established across different specialties [5-10]. However, a recent study in the UK found that there are important sources of systematic bias influencing these multisource assessments, such as specialty and whether or not a doctor works in a locum capacity [11]. This implies that a MSF score given to a doctor might be more affected by sociodemographic variables of the respondent than by the doctors' true performance, which should be investigated across different MSF settings [12].

In addition, it has recently been underlined that instruments validated in one setting should not be used in new settings without revalidation and updating since validation is an ongoing process, not a one-time event [13]. Hence, given the significance of the judgments made, in terms of both patient safety and the usefulness of MSF for physicians' professional development, it is essential to develop and validate assessment instruments in new settings as rigorously as possible. This paper reports on the validation study of three MSF measurement instruments used in in the Netherlands, namely peer completed, co-worker-completed and patient-completed. Specifically, this paper addresses three core aims, namely: (1) the initial psychometric properties of three new instruments based on existing MSF instruments and the influence of potential sociodemographic variables, (2) the correlation between physician self-evaluation and other raters' evaluations, (3) the number of evaluations needed per physician for reliable assessments.

## Methods

### Ethics statement

The study was given expedited approval by the Central Committee on Research Involving Human Subjects (known by its Dutch initials, CCMO), the local institutional review board.

### MSF system in the Netherlands

The MSF system in the Netherlands consists of feedback from physician colleagues (peers), co-workers and patients. This is combined with a reflective portfolio and an interview with a trained mentor (a colleague from a different specialty based in the same hospital) to facilitate the acceptance of feedback and, ultimately, improved performance. To guide performance, the mentor helps physicians interpret the feedback and critically analyze their performance making use of the feedback. In 2007, as part of a larger physicians' performance project, the MSF system was launched in three hospitals for physician performance assessment and a pilot study established its feasibility [14]. Subsequently, the MSF system was adopted by 23 other hospitals. The MSF process is managed electronically by an independent web service. Physicians are invited via e-mail and asked to complete a self-evaluation form and nominate up to 16 raters (8 peers and 8 co-workers). All raters except patients are contacted by e-mail and are asked to complete a questionnaire via a dedicated web portal protected by a password login. The web service automatically sends reminders to non-respondents after 2 weeks. Data collection from patients takes place via paper questionnaires which are handed out by the receptionist to consecutive patients attending the outpatient clinic of the physician participating. Patients are asked to complete the questionnaire after the consultation and anonymity of the questionnaire is explained by the receptionist. Patients can post the completed form in a sealed box after the consultation. The web-based service provides electronic feedback reports to the mentor and physician to be discussed face-to-face in a personal interview. The report contains global overall graphic and detailed numeric outcomes of the peers, co-workers and patients' evaluations as well as the self-evaluation. Free text comments (answers from raters to open questions about the strengths of the physicians and opportunities for improvement) are also provided at the end of the MSF report.

### MSF instrument and development

There were two distinct stages of instrument development as part of the validation study. The two stages are described below.

#### Content generation and content validity

The research committee (5 members) drafted a questionnaire and drew on previously developed MSF instruments for medical and surgical specialties in Canada owned by the College of Physicians & Surgeons of Alberta [2]. The 20 items of the patient questionnaire that concerned management of the practice (such as performance of staff at the outpatient clinic) were removed as the aim of the project was to measure physicians' professional performance and those items are the subject of another system [15]. Two researchers translated the items of the questionnaires from English to Dutch with the help of a native English speaker. A backward translation-check was performed by an independent third person. Next, content validity was established in a small study. Fifteen physicians, ten co-workers and ten patients were asked to rate the relevance and clarity of questions on a 1 to 4 scale. (1 = not relevant/not clear, 4 = very relevant/very clear). The accepted norm for inclusion of an item in its current format was set at 70 percent of respondents agreed on relevance (a score of 3 or 4). An item was reformulated if less than 70 percent or respondents agreed on clarity (a score of 3 or 4). For the peers' and co-workers' questionnaires, all original items were found to be relevant; 6 items on the peer questionnaire needed reformulation for clarity. Two items were removed from the patient questionnaires as they were perceived as irrelevant for the Dutch context and eight items of the patient questionnaire needed reformulation for clarity.

#### Pilot field testing

In total, 45 physicians participated in a pilot test to investigate the feasibility of the system and appropriateness of items. The feasibility results are described elsewhere [14]. The *appropriateness *of items was evaluated through the item-response frequencies. Raters had the choice of selecting 'unable to evaluate' for each item. An item was judged suitable for the MSF questionnaire if at least 60 percent of the raters (peers, co-workers or patients) responded to the item. After analysis of items with a > 40 percent category of 'unable to evaluate', five items were removed from the peer questionnaire and two items were removed from the patient questionnaire.

#### Final MSF system

The final MSF system used in the study and presented in this paper comprised three questionnaires, each prefaced by an introduction. The peer questionnaire consisted of 33 performance items; the co-worker and patient questionnaires included 22 and 18 items respectively. All items invited responses on a 9-point Likert type scale: (1 = completely disagree, 5 = neutral, 9 = completely agree). For every item, raters had the option to fill in: 'unable to evaluate'. In addition, all raters were asked to fill in two open questions for narrative feedback, listing the strengths of individual physicians and formulating concrete suggestions for improvement.

### Study design, population and setting

This observational validation study on the use of three MSF instruments in actual practice was set in 26 non-academic hospitals in the Netherlands, including both surgical and medical specialties. For several specialties such as anesthesiology and radiology specialty specific instruments were developed and therefore excluded from our study [5,16]. All physicians who completed the interview with a mentor were approached to participate. No financial incentives were provided and participants could withdraw from the study at any time without penalty. Participating hospital-based physicians consented to provide their anonymous data for research analysis. We aimed to obtain a large sample with sufficient data (more than 100 physicians) to allow an assessment of the performance of the questionnaires in line with recognized best practice [13]. Data collection took place in the period September 2008 to July 2010. The analysis presented in this paper used anonymised datasets derived from this volunteer sample.

### Statistical analysis

For the final instrument, we first removed all items for which the response 'unable to evaluate or rate' was more than 15 percent. Furthermore, the data of respondents who responded to less than 50 percent of all items were not included in the analysis. To address the first objective of this study, that is, to investigate the psychometric properties of the MSF instruments, we conducted principal components analysis, reliability coefficient, item-total scale correlation, and interscale correlation analyses [13,17]. For item reduction and exploring the factor structure of the instruments, we conducted principal components analysis with an extraction criterion of Eigenvalue > 1 and with varimax rotation. Items were grouped under the factor where they displayed the highest factor loading. Subsequently, the factor structure was subjected to reliability analysis using Cronbach's alpha. We considered a Cronbach's alpha of at least 0.70 as an indication of satisfactory internal consistency reliability of each factor [18]. We also checked for homogeneity of factors by examining the item-total correlations, while correcting for item overlap [13]. We considered an item-total correlation coefficient of 0.3 or more adequate evidence of homogeneity, hence reliability. We checked for overlap between factors by estimating inter-scale correlations using Pearsons' correlation coefficient. An inter-scale correlation of less than 0.70 was taken as a satisfactory indication of non-redundancy [17,19]. To quantify the potential influences on the physicians' ratings, we built a model which accounted for the clustering effect of the individual physician and the bias with which an individual rater (peer, co-worker or patient) rated the physician. Therefore, we used a linear mixed-effects model to look at the adjusted estimate of each variable while correcting for the nesting or clustering of raters within physicians. As predictor variables, we included gender of the rater, length of the professional relationship between the rater and physician, specialty, work experience of the physician, gender of the physician, and physician group membership. To address the second research objective of our study, that is, the relationships between the four (peer, co-worker, patient and self) measurement perspectives, we used Pearsons' correlation coefficient using the mean score of all items. To address our final research objective, the number of evaluations needed per physician to establish the reliability of assessments, we used classical test theory and generalisability theory methods. We assumed that, for each instrument, the ratio of the sample size to the reliability coefficient would be approximately constant across combinations of sample size and associated reliability coefficients in large study samples. Therefore, if any new pre-specified reliability coefficient was less than or equal to that observed in our study, then the required number of raters' evaluations per physician should resemble that observed in our study [13,20,21]. To check this assumption using our data, we re-estimated the reliability for the different sample sizes predicted by the measure of precision and spread of scores, in line with other studies [22]. We calculated 95% CIs by multiplying the SEM (standard error of measurement) by 1.96 and adding and subtracting this from the mean rating [22]. "This CI can then be placed around the mean score, providing a measure of precision and, therefore, the reliability that can be attributed to each mean score based on the number of individual scores contributing to it" *[verbatim quote] *[22].

## Results

### Study participants

A total of 146 physicians participated in the study. In total 864 peers (a mean of 6.5 per physician), 894 co-workers (a mean of 6.7 per physician) and 1890 patients (a mean of 15 per physician) rated the physicians. Forty percent of the physician participants was female. This is in line with the percentage of female hospital based physicians in the Netherlands. The mean number of years since first registration of the physicians was 13.6 years, (minimum 2 years; maximum 35 years; standard deviation 8.4 years).

### Mean ratings and missing data

Peers scored physicians highest on the items 'responsibility for patients' (mean = 8.67) and 'responsibility for own professional actions' (mean = 8.64). Peers provided the lowest ratings for the item 'research activities' (mean = 7.67) and 'evaluating literature' (mean = 7.96). When aggregated for the individual physician, the mean rating given by peers was 8.37, ranging from 7.67 (min 1 max 9 SD 1.75) to 8.69 (min 2 max 9 SD 0.70). All items were positively skewed. Co-workers rated physicians highest on 'responsibility for professional actions' (mean = 8.64) and lowest on 'verbal communication with co-workers' (mean = 7.78). Patients rated physicians highest on 'respect' (8.54) and gave physicians the lowest rating for 'asking details about personal life' (mean = 7.72). Missing data (unable to comment) ranged from 4 percent of co-workers' responding on the item 'collaborates with physician colleagues' to 38.9 percent of peers evaluating physicians' performance on 'participates adequately in research activities'. On average, per item, the mean of missing data was 19.3 percent for peers, 10 percent for co-workers' responses and 17.7 percent for patients. All mean scores of items are summarized in Table 1, 2 and 3.

**Table 1:** Factors derived from the principal components analysis of peers' ratings

Factors and items	Mean score [SD]	Factor loadings on primary factor	Internal consistency reliability	Corrected item-total correlations
*Collaboration and self-insight (42% of variance)*	8.47 [1.09]		0.900	

Communicates effectively with other health care professionals	8.23 [1.07]	0.581		0.655

Collaborates with physician colleagues	8.56 [0.91]	0.841		0.795

Accepts feedback provided	8.38 [1.06]	0.748		0.711

Recognizes his/her own limitations	8.13 [1.19]	0.643		0.702

Participates effectively as a member of the health care team	8.40 [1.21]	0.631		0.700

Exhibits professional behavior towards physician colleagues	8.61 [0.85]	0.779		0.750

If a member of my own family needed care I would recommend this physician	8.59 [0.84]	0.760		0.761

*Clinical performance (8% of variance)*	8.40 [0.79]		0.900	

Performs technical procedures skillfully	8.45 [0.96]	0.638		0.615

Selects diagnostic tests appropriately	8.38 [0.93]	0.739		0.758

Critically assesses diagnostic information	8.44 [0.93]	0.763		0.823

Makes the correct diagnosis following consultation	8.43 [0.88]	0.780		0.822

Selects appropriate treatments	8.41 [0.93]	0.650		0.791

Accepts responsibility for own professional actions	8.4 [0.91]	0.452		0.629

*Coordination and continuity* *(5% of variance)*	8.47 [0.73]		0.851	

Handles transfer of care appropriately	8.37 [1.03]	0.727		0.697

Maintains confidentiality of patients and their families	8.57 [0.89]	0.660		0.601

Provides a clear understanding about who is responsible for the continuing care of patients	8.35 [0.94]	0.632		0.717

Co-ordinates care effectively for patients with other health care professionals and physicians	8.44 [0.89]	0.609		0.684

Maintains quality medical records	8.13 [1.23]	0.456		0.547

Manages patients with complex problems	8.46 [0.88]	0.632		0.669

*Practice based learning and improvement* (4% of variance)	8.12 [1.13]		0.813	

Contributes to quality improvement programs and practice guidelines	8.22 [1.26]	0.652		0.726

Teaches adequately medical colleagues and co-workers	7.97 [1.42]	0.652		0.728

Participates adequately in research activities	7.67 [1.52]	0.599		0.739

Critically evaluates the medical literature	7.96 [1.36]	0.655		0.725

*Emergency medicine* *(4% of variance)*	8.38 [0.85]		0.767	

Gives priority to urgent requests	8.46 [0.93]	0.660		0.634

Handles emergency situations effectively	8.49 [0.91]	0.703		0.631

Manages own stress effectively	8.22 [1.12]	0.564		0.549

*Time-management and responsibility* *(4% of variance)*	8.69 [1.30]		0.770	

Handles requests for consultation in a timely manner	8.40 [1.00]	0.749		0.645

Advises referring physician if referral request is outside the scope of his/her practice	8.53 [0.87]	0.550		0.576

Assumes appropriate responsibility for patients	8.67 [0.69]	0.527		0.539

Provides timely information to referring physicians about mutual patients	8.28 [0.13]	0.690		0.608

**Table 2:** Factors derived from the principal components analysis of co-workers' ratings

Factors and items	Mean score [SD]	Factor loadings on primary factor	Internal consistency reliability	Corrected item-total correlations
*Relationship with other health care professionals (57% of variance)*	8.07 [1.11]		.925	

Is able to verbally communicate effectively with other health care professionals	8.10 [1.21]	0.691		0.757

Is courteous to co-workers	8.35 [1.11]	0.760		0.763

Respects the professional knowledge and skills of co-workers	8.31 [1.10]	0.782		0.765

Collaborates well with co-workers	8.31 [1.10]	0.811		0.848

Is accessible for appropriate communication about patients	8.37 [1.06]	0.611		0.728

Participates effectively as a member of the health care team	8.28 [1.13]	0.660		0.766

This physician presents him/herself in a professional manner	8.59 [0.92]	0.574		0.740

*Communication with patients (7% of variance)*	8.03 [1.07]		.900	

Communicates effectively with patients	8.21 [1.15]	0.830		0.794

Communicates effectively with families	8.11 [1.24]	0.818		0.764

Shows compassion to patients and their families	8.36 [1.07]	0.721		0.812

Is courteous to patients and their families	8.56 [0.89]	0.656		0.772

Respects the rights of patients to make informed decisions	8.46 [0.96]	0.578		0.706

Is reasonably accessible to patients	8.28 [1.08]	0.579		0.700

*Patient care (6% of variance)*	8.29 [1.06]		.830	

Accepts responsibility for patient care	8.64 [0.72]	0.748		0.720

Maintains confidentiality of patients	8.69 [0.77]	0.711		0.613

Accepts responsibility for professional actions	8.64 [0.86]	0.781		0.773

Responds appropriately in emergency situations	8.40 [1.11]	0.643		0.586

**Table 3:** Factors derived from the principal components analysis of patients' ratings

Factor and items	**Mean score[SD]**	Factor loadings	Internal consistency reliability	Corrected item-total correlations
*Patient-centeredness (60% of variance)*			.959	

Explained my illness or concern to me clearly	8.30 [1.32]	0.792		0.792

Spends enough time with me	8.29 [1.38]	0.825		0.781

Shows interest in my problems	8.25 [1.43]	0.840		0.806

Answers my questions well	8.33 [1.32]	0.873		0.827

Treats me with respect	8.54 [1.02]	0.810		0.763

Shows compassion	8.10 [1.53]	0.804		0.770

I would go back to this physician	8.50 [1.23]	0.819		0.803

I would recommend this physician to others	8.43 [1.34]	0.828		0.823

Explains my treatment choices or options	8.11 [1.48]	0.771		0.769

Tells me how and when to take my medicine	8.00 [1.59]	0.646		0.722

Explains clearly different steps of my treatment plan (including risks and benefits)	8.06 [1.53]	0.763		0.779

Asks details about my personal history, when appropriate	7.72 [1.83]	0.664		0.659

Explains my physical exam clearly	8.13 [1.49]	0.799		0.750

Asks permission for some treatments or exams	8.03 [1.60]	0.689		0.697

Explains clearly what could be done in unsuspected circumstances, such as fever, illness or changes in my complaints	7.84 [1.70]	0.757		0.754

Tells me what to do if my problems do not get better	7.90 [1.23]	0.785		0.787

Makes sure that my other caregivers are well informed	7.94 [1.68]	0.670		0.650

### Dimension structure and reliability of the dimensions

Factor loadings from principal components analysis of the peer ratings, yielded 6 factors with an Eigen value greater than 1, in total explaining 67 percent of variance. The factors comprised: collaboration and self-insight, clinical performance, coordination & continuity, practice based learning and improvement, emergency medicine, time management & responsibility. Due to low factor loadings, three items were eliminated. The six factors were highly consistent with the structure of the questionnaire, as defined by items having a factor loading greater than 0.4 (Table 1). Principal components analysis of the co-worker instrument revealed a 3-factor structure explaining 70 percent of variance. Because of low factor loadings and high frequency of 'unable to evaluate', five items were removed from the instrument. Factors included: relationship with other healthcare professionals, communication with patients and patient care. The principal components analysis of the patient ratings yielded a 1-factor structure explaining 60 percent of the total variance. Cronbach's alphas were high for peers', co-workers' and patients' composite factors, ranging from 0.77 to 0.95. (Table 1, 2 and 3) Item-total correlations yielded homogeneity within composite factors. Cronbach's alpha for the peer, co-worker and patient questionnaires were 0.95, 0.95 and 0.94 respectively, indicating good internal consistency and reliability of the questionnaires. Item-total correlations yielded homogeneity within composite factors. Inter-scale correlations were positive and < 0.7, indicating that all the factors of the three instruments were distinct. (see Table 4 and 5)

**Table 4:** Pearson correlation coefficient between peers' factors

	Collaboration	Clinical performance	Practice based learning and improvement	Coordination and continuity	Responsibility and time-management	Emergency medicine
Collaboration	1.000	0.499**	0.451**	0.295**	0.459	0.437

Clinical performance		1.000	0.551	0.432**	0.383	0.408

Practice based learning and improvement			1.000	0.357**	0.445	0.400**

Coordination and continuity				1.000	0.338	0.343**

Responsibility and time-management					1.000	0.328**

Emergency medicine						1.000

** correlation is significant at 0.01 level

**Table 5:** Pearson correlation coefficient between co-workers' factors

	Relationship with healthcare professionals	Communications with patients	Professionalism
Relationship with healthcare professionals	1.000	0.667**	0.537**

Communications with patients		1.000	0.574**

Patient care			1.000

**correlation is significant at 0.01 level

### Sociodemographic variables influencing rating

The linear mixed model showed that membership of the same physician group was positively correlated with the overall rating given to colleagues (beta = 0.153, p < 0.01). There was a small but significant influence of physicians' work experience, showing that physicians with more experience tend to be rated lower by peers (beta = -0.008, p < 0.05) and co-workers (Beta = -0.012, p < 0.05). These two biasing factors accounted for 2 percent of variance in ratings. Across co-worker assessors there was a significant difference in scores on the basis of gender, showing that male co-workers tend to score physicians lower compared to female co-workers. (Beta = -0.200, p < 0.001). This factor explained 2 percent of variance. We found no statistical effect of the length of the relationship of the co-workers and peers with the physician. The patients' age was positively correlated with the ratings provided to the physician (Beta = 0.005, p < 0.001). Finally, we found no statistical influence of patients' gender. The model for patient ratings accounted for only 3 percent of the variance in ratings. Parameter estimates of the various biasing factors are summarized in Table 6.

**Table 6:** Effects of raters' characteristics and physicians' characteristics on overall mean scores

	Overall rating peers		Overall rating co-workers		Overall rating patients	
	Parameter estimated coefficient* [SE]	**P-value**	Parameter estimated coefficient* [SE]	**P-value**	**Parameter** estimated coefficient* [SE]	**P-value**

*Physicians'* *characteristics*						

Male (reference: female)	-.0137 [.065]	.832	-.019 [.091]	.838	-.049 [.091]	.591

Years of experience	-.008 [.004]	**.043**	-.012 [.005]	**.029**	.003 [.005]	.598

Surgery	ref.		ref.		ref.	

Internal medicine	.069 [.064]	.287	.139 [.094]	.140	.096 [.088]	.280

*Raters' characteristics*						

Female	ref.		ref.		ref.	

Male	-.002 [.051]	.974	-.200 [.071]	**.005**	-.055 [.062]	.378

Age					.005 [.002]	**.002**

*Relation*						

Membership of the same specialist group	.153 [.049]	**.002**				

Working together: < 6 months	-.115 [.179]	.523	.046 [.185]	.804		

Working together: > 6 months and < 1 year	.042 [.111]	.702	-.036 [.122]	.770		

Working together > 1 year	ref.	ref.				

*p < 0.05

### Relationship between the different ratings

Self-ratings were not correlated with the peer ratings, co-worker ratings or patient ratings. Ratings from peers, co-workers and patients in the MSF procedure appeared to be correlated. Peer ratings were positively associated with the patient ratings (r = 0.214, p < 0.01). The correlation between the peer ratings and the co-worker ratings was significant as well (r = 0.352, p < 0.01). Finally, co-worker ratings appeared to be positively associated with patient ratings. (r = 0.220, p < 0.01). Overall, all correlations appeared to be small. Table 7 shows the correlations between the mean scores for self ratings, peer ratings, co-worker ratings and patient ratings.

**Table 7:** Pearsons' correlation coefficients between the ratings of four measurements perspectives: self, colleagues, co-workers and patients

	Self rating	Medical colleagues' ratings	Co-workers' ratings	Patient ratings'
Self rating	1.000	0.062	0.082	0.067

Medical colleagues' ratings		1.000	0.352*	0.214*

Co-workers' ratings			1.000	0.220*

Patient ratings'				1.000

* correlation is significant at 0.05 level

### Determining the minimum sample size required

Following the methods of a previous work [21], we estimated the minimum number of evaluations per physician needed to achieve specified reliability coefficients: assuming a reliability coefficient of 0.60, ratings from 4 peers, 4 co-workers and 9 patients would be required for reliable measurement. When a stricter reliability coefficient of 0.70 was applied, as many as 5 peers, 5 co-workers and 11 patients evaluating each physician would be required. Table 8 summarizes the number of raters needed for reliable results. Reliability calculations based on 95% CIs and the residual component score showed that, with 5 peers, 5 co-workers and 11 patients, none of the physicians scored less than the criterion standard, in our case 6.0 on a 9-point standard. The various variance components (true variance and residual variance) necessary for this calculation are provided in Table 9.

**Table 8:** Number of colleagues, co-workers and patients' evaluations needed per physician for reliable evaluation of physicians' professional performance for different reliability coefficients

	Reliability coefficient of 0.60	Reliability coefficient of 0.70	Reliability coefficient of 0.80
Peers	4	5	5

Co-workers	4	5	6

Patients	9	11	12

**Table 9:** Variance components for the three different groups of raters

	True variance	Residual variance
Peers	0.06	0.42

Co-workers	0.16	0.45

Patients	0.093	1.07

## Discussion

### Main findings

This study shows that the adapted Canadian MSF tool, incorporating peer, co-worker and patient feedback questionnaires is reliable and valid for hospital-based physicians (surgical and medical). We found robust factor structures with good internal consistency across the three instruments. Our study demonstrates that little of the variance in performance could be explained by factors, such as gender of the rater and length of the relationship with the rater, that were beyond the physicians' control. Physicians were rated more positively by members of their physician group, but this accounted for only two percent of variance in ratings. Individual reliable feedback reports could be generated with a minimum of 5 evaluations of peers, 5 co-workers and 11 patients respectively.

### Explanation and interpretation

Our findings provide strong empirical support for the reliability and validity of the results obtained from the three MSF instruments for physicians' performance evaluation. The results of the psychometric analyses for the three MSF instruments indicate that we could tap into multiple factors per questionnaire. For the peer instrument, our factor analysis suggested a 6-dimensional structure. These findings do not support the 4-dimensional structure found in earlier research of the original instruments by Violato and Lockyer. Other studies of instruments used for MSF by Archer et al. [23] and Ramsey et al. [24] assess two generic factors; labeled as clinical and psychosocial qualities. Our findings do not confirm the suggestions made in earlier studies that found only two generic factors [20] Those researchers argue that in MSF evaluations, the halo effect -which is the tendency to give global impressions- and stereotyping exist [25]. This does not seem to apply to Dutch hospital physicians evaluating colleagues. Physicians seem to be able to distinguish between different aspects of professional performance instead of giving global impressions concerning the clinical performance and humanistic qualities.

However, our results underline that peers, co-workers and patients tend to answer on the upper end of the scale, also known as positive skewness. It is not yet clear whether this is the result of the fact that questions are in general formulated with a positive tone or for example because of the nature of the study (it is not a daily scenario). Other studies show similar results [23,24]. The interpretation of these scores might lead to limited directions for change. Our finding that self-ratings using MSF are not related with ratings made by peers, co-workers and patients is consistent with the current literature on self-assessment and justifies the introduction of MSF for the evaluation of physicians' professional performance [1]. However, we found support for significant correlations between ratings of peers, co-workers and patients. They can be considered as three independent groups of raters, representing different perspectives, thus supporting the existence of concurrent validity. Similar with other MSF instruments, we have not formally tested the criterion validity of instruments, because a separate gold standard test is lacking [11].

Previous studies with original MSF-questionnaires in Canada demonstrated that 8 peer evaluations,7 co-worker evaluations and 25 patient evaluations are required to produce reliable results [7] while studies in the UK amongst residents found that 4 evaluations are needed [23]. Compared to Canada, in the Netherlands less evaluations are necessary to achieve reliable results. Potentially, teams and physician groups in the Netherlands are smaller, increasing the interdependence of work as well as opportunities of observing colleagues' performance [26].

### Strengths and limitations

This study was restricted to a self-selected sample of physicians receiving feedback. It is likely that those who agreed to participate were reasonably confident about their own standards of practice and the sample may have been skewed towards good performance. The mean scores, however, are similar to scores reported by other comparable instruments that were also skewed to good performance [24]. Second, we could use only 80 percent of peer responses due to missing values on one or more items. Future work should investigate whether missing values are indicative of the tendency to avoid a negative judgment. Third, participant physicians were asked to distribute the survey to consecutive patients at the outpatient clinic but we were not able to check if this was correctly executed for all participants. Fourth, because of the cross-sectional design of this study, an assessment of intra-rater (intra-colleague or intra-co-worker) or test-retest reliability was not possible. Further work on the temporal stability of responses of the questionnaires is warranted. Finally, the data being anonymous, the hospital and specialist group specialists were based in were not available for analysis. It would have been interesting to investigate the effects of various hospitals and specialty groups on reported change as these factors have been found to be important determinants in previous studies [11].

### Implications for practice and research

This study established the validity and reliability of MSF for hospital-based physicians in the Netherlands. Although it cannot be expected that one single tool can guide improvement for all physicians, it offers Dutch physicians feedback about their performance. MSF in the Netherlands has been designed and tested for formative purposes. The purpose is to give feedback to physicians so that they can steer their professional development plans towards achieving performance excellence [27]. Reliable results are achieved with 5 peer, 5 co-workers and 11 patient raters, which underscores that implementation is attainable in academic and non-academic hospitals. With respect to the positive skewness of the results of the questionnaires, presumably the idea of visualizing the outcomes into 'excellent ratings' versus 'sufficient ratings' and 'lower ratings' presents deficiencies more clearly. This approach might increase the educational potential of MSF [28].

We did not test the possibility to use the results of our study to draw conclusions about the ability to detect physicians whose performance might be below standard. In view of the positive skewness of results and the fact that criterion validity is not yet tested, we consider this as an undesirable development. We consider this study a starting point for further research. As a result we do not claim the items presented in the tables to be the final version, because a validation process should be ongoing. Furthermore, additional work is required to further establish the validity of the instruments. We agree with Archer et al. that MSF is unlikely to be successful without robust regular quality assurance to establish and maintain validity including reliability [22]. Further validity of the factors could be tested by comparing scores with observational studies of actual performance requiring external teams of observers or mystery patients.

## Conclusions

This study supports the reliability and validity of peer, co-worker and patient completed instruments underlying the MSF system for hospital based physicians in the Netherlands. Reliable individual feedback reports can be generated based on a minimum of respectively five, five and 11 evaluations. Physicians may use their individual feedback reports for reflection and designing personal development plans.

## Competing interests

The authors declare that they have no competing interests.

## Authors' contributions

Conceived and designed the experiments: KO KML HCW. Analyzed the data: KO KML JC OAA. Contributed reagents/materials/analysis tools: KO JC OAA. Wrote the paper: KO. Editing and reviewing the manuscript: KML HCW PRTMG OAA JC. All authors read and approved the final manuscript.

## Pre-publication history

The pre-publication history for this paper can be accessed here:

http://www.biomedcentral.com/1472-6963/12/80/prepub
